# Atrial fibrillation in cancer patients who develop stroke

**DOI:** 10.1186/s40959-022-00137-y

**Published:** 2022-05-18

**Authors:** Alia Khamis, Ala Emad Shaban, Tamer Salhab Altamimi, Zakaria Walid Shkoukani, Ismail Hamam

**Affiliations:** 1 Office of Scientific Affairs and Research, King Hussein Cancer Center, Amman, Jordan; 2 Department of Internal Medicine, King Hussein Cancer Center, Queen Rania Al-Abdullah Street, P.O.Box 1269, Amman, Al-Jubaiha 11941 Jordan

**Keywords:** Ischemic stroke, Transient ischemic attack, Cancer, Atrial fibrillation

## Abstract

**Background:**

Acute ischemic stroke (Stroke) and transient ischemic attacks (TIA) are known complications in cancer patients and those with atrial fibrillation (AF). The role AF plays in Stroke/TIA in the setting of cancer is unclear. The purpose of this study was to assess the relationship between AF and Stroke/TIA in cancer patients.

**Methods:**

We conducted a case-control study comparing all patients who developed Stroke/TIA from 2014 to 2019 following a cancer diagnosis at King Hussein Cancer Center (KHCC), matched to Stroke/TIA-free controls based on age, gender, and cancer site.

**Results:**

Two hundred seventy-two patients were included (136 per group). The mean age was 63.95 ± 13.06 and 57% were females. The Stroke/TIA group had more AF at the time of event (14% vs. 4%, OR: 4.25, 95%-CI: 1.39 - 17.36) and had a larger proportion of death on study conclusion (OR: 9.4, 95%-CI: 3.74 - 23.64). On conditional logistic regression, patients in the Stroke/TIA group had higher odds of: AF (OR: 7.93, 95%-CI: 1.6 – 39.18), ischemic stroke before cancer diagnosis (OR: 9.18, 95%-CI: 2.66 – 31.74), being on active cancer treatment (OR: 3.11, 95%-CI: 1.46 – 6.62), dyslipidemia (OR: 3.78, 95%-CI: 1.32 – 10.82), and renal disease (OR: 4.25, 95%-CI: 1.55 – 11.63). On another conditional logistic regression model built to assess the role of the CHA_2_DS_2_-VASc score, a score of >=2 in males and >=3 in females significantly increased the risk of developing Stroke/TIA in cancer patients (OR: 2.45, 95%-CI: 1.08 - 5.58).

**Conclusion:**

AF, previous ischemic stroke, active cancer treatment, dyslipidemia, and renal disease are independent risk factors for Stroke/TIA and a higher CHA_2_DS_2_-VASc score significantly increases the risk in cancer patients regardless of AF.

**Supplementary Information:**

The online version contains supplementary material available at 10.1186/s40959-022-00137-y.

## Introduction

Cerebrovascular diseases (CVD) are common in cancer patients and are associated with a worse prognosis [1]. CVD in cancer patients occurs at a rate of approximately 15% with ischemic and hemorrhagic strokes having nearly equal rates [2, 3]. Stroke might be an underlying comorbidity or might follow a cancer diagnosis [4, 5]. In addition, the prevalence of cancer in stroke patients is higher than that of the general population [6]. In an analysis of more than 250,000 patients with cancer paired with an equal number without cancer, the cumulative incidence of stroke at 6 months was 3% and 1.6%, respectively: presenting a two-fold increase in stroke risk over the general population [7, 8].

Atrial fibrillation (AF) is also common in cancer patients with approximately 2-5% of cancer patients having AF at the time of diagnosis [9–11]. In addition, new-onset AF has a higher incidence in cancer patients compared to the general population [12]. Higher rates of AF in cancer patients might be explained by the shared risk factors with the general population in addition to cancer-induced inflammation, anemia, hypoxemia, surgery, and sepsis [5, 11, 13].

Acute ischemic stroke (Stroke) and transient ischemic attacks (TIA) are known complications in both cancer patients and patients with AF but only until recently, the relationship observed between AF and cancer was limited to epidemiologic data showing that the diagnosis of either one increases the odds of being diagnosed with the other. Nonetheless, the risk of stroke in patients with concurrent cancer and AF is unclear and still needs more evidence.

The risk of thromboembolic events is assessed in each patient suffering from AF and the CHA_2_DS_2_-VASc score is the most common scheme used to do so. However, since cancer patients are generally assumed to have an increased risk of thromboembolism, this might affect the risk of stroke in patients with cancer and AF. Furthermore, patients with active cancer were not included in trials that validated CHA_2_DS_2_-VASc as a stroke prediction score [10, 14, 15] rendering the role of this score in predicting stroke risk in cancer patients unclear.

In this study, we aim to evaluate the role of AF in the development of Stroke/TIA in cancer patients. Other risk factors of Stroke/TIA are also investigated. Finally, the role of CHA_2_DS_2_-VASc in predicting Stroke/TIA in cancer patients is studied.

## Methods

### Ethical conduct of research

This study was approved by the Institutional Review Board committee at King Hussein Cancer Center. All procedures performed in this study involving human participants were in accordance with the ethical standards of the institutional research committee and with the 1964 Helsinki declaration and its later amendments or comparable ethical standards. This article does not contain any studies on animals performed by any of the authors. Informed consent was waived by the Institutional Review Board committee due to the retrospective nature of the study.

### Study protocol

We conducted a matched case-control study. Cases who developed Stroke/TIA from 2014 to 2019 following a cancer diagnosis at King Hussein Cancer Center were matched to the controls who had cancer but did not develop Stroke/TIA up to the last day of follow up. The cases were individually matched to the controls on a 1:1 ratio based on age, gender, and site of cancer. All relevant patient information was retrieved retrospectively from electronic medical records.

All patient records were screened using automatic search. Cases were consecutively included if they developed Stroke/TIA between 2014 and 2019. Stroke/TIA diagnosis was either made by brain imaging or clinical evaluation at the time of the event by the following physician inside the center. Controls were retrieved based on the matched characteristics from the database.

Patients were not included if they had hemorrhagic stroke, infarction caused by brain metastasis, multiple concurrent cancers, or unconfirmed Stroke/TIA diagnosis.

Collected data included details related to age, gender, cancer type, cancer stage, cardiac rhythm on presentation, comorbidities, chemotherapy, radiotherapy, hormonal therapy, cancer-related surgery, use of anticoagulants, anti-platelets, and outcome. In addition, this information was utilized to calculate the CHA_2_DS_2_-VASc and HAS-BLED scores for each participant. Definitions of each variable are presented in the supplement.

The primary outcome of our study was to examine the presence of AF as an independent risk factor for Stroke/TIA in cancer patients. The secondary outcomes included examination of the associations of clinical characteristics, treatment, and risk-stratifying scores with Stroke/TIA in cancer patients.

### Statistical analysis

Statistical analysis was conducted using RStudio Version 1.3.1093. Categorical variables were presented as frequencies and percentages while quantitative variables were expressed as median and interquartile range (IQR). Due to the study design of 1:1 individual matching of cases to controls, matched analyses were adapted. Univariate analyses were carried out to assess the differences/associations between Stroke/TIA patients and their matched controls using the Wilcoxon test for continuous variables and McNemar's test with continuity correction or exact test for categorical variables in 2x2 tables. Categorical variables with 3x3 tables were analyzed using McNemar-Bowker Test. A level of significance < 0.05 was set for all univariate analyses. In addition, a subgroup analysis of specific cancer types was performed using an exact test. Next, a conditional logistic regression model was built including variables that had a *p*-value < 0.2 in the univariate analyses. Variables with a sample size below 25 were excluded from the model. In addition, variance inflation factor (VIF) level of > 5 and tolerance of < 0.2 were set to detect multicollinear variables; however, none of the variables had VIF higher than 5. All the variables which fit the inclusion criteria of the model were found to be categorical. Furthermore, another conditional logistic regression model was built to assess the effect of the CHA_2_DS_2_-VASc score. Variables that were included in computing CHA_2_DS_2_-VASc score were excluded from the model. A level of significance < 0.05 was set for both regression models.

## Results

A total of 272 cancer patients divided into 136 Stroke/TIA patients were matched to 136 controls without Stroke/TIA. Table 1 summarizes the matched characteristics between the 2 groups, while Table 2 summarizes univariate associations.

**Table 1:** Matched characteristics of patients

Variable	Total (***n***= 272)	Stroke/TIA (***n***=136)	No Stroke/TIA (***n***=136)
Age	63.95 ± 13.06	63.93 ± 13.18	63.98 ± 12.99
Male	118 (43%)	59 (43%)	59 (43%)
Cancer Site			
Gastrointestinal Tract	66 (24%)	33 (24%)	33 (24%)
Breast	40 (15%)	20 (15%)	20 (15%)
Lung and Pleura	40 (15%)	20 (15%)	20 (15%)
Urological	34 (12%)	17 (12%)	17 (12%)
Head and Neck	30 (11%)	15 (11%)	15 (11%)
Hematological	28 (10%)	14 (10%)	14 (10%)
Gynecological	22 (8%)	11 (8%)	11 (8%)
Intracranial	8 (3%)	4 (3%)	4 (3%)
Bone	2 (1%)	1 (1%)	1 (1%)
Others	2 (1%)	1 (1%)	1 (1%)

**Table 2:** Univariate analysis of patients’ clinical characteristics

Variable	Total (***n*** = 272)	Stroke/TIA (***n*** = 136)	No Stroke/TIA (***n*** = 136)	***p***-value
Cancer Stage				
Localized	59 (22%)	26 (19%)	33 (24%)	.320
Regional	49 (18%)	21 (15%)	28 (21%)	
Distant	125 (46%)	69 (51%)	56 (41%)	
Unstageable	39 (14%)	20 (15%)	19 (14%)	
Cancer-Specific Management				
Active Cancer Treatment at The Time of Event (Stroke/TIA)	94 (35%)	60 (44%)	34 (25%)	.002
History of Chemotherapy	161 (59%)	83 (61%)	78 (57%)	.600
History of Hormonal Therapy	32 (12%)	13 (10%)	19 (14%)	.140
History of Radiotherapy	108 (40%)	56 (41%)	52 (38%)	.670
History of Cancer-Related Surgery	125/ 266 (47%)	57 (42%)	68/ 130 (52%)	.070
Atrial Fibrillation (New onset AF or baseline AF)	25 (9%)	19 (14%)	6 (4%)	.007
Comorbidities				
Any	246 (90%)	131 (96%)	115 (85%)	.002
Hypertension	158 (58%)	88 (65%)	70 (52%)	.030
Diabetes Mellitus	111 (41%)	64 (47%)	47 (35%)	.040
Smoking	Smoker = 88 (32%) Ex-smoker = 43 (16%)	Smoker = 45 (33%) Ex-Smoker = 22 (16%)	Smoker = 43 (32%) Ex-smoker = 21 (15%)	.950
Dyslipidemia	68 (25%)	46 (34%)	22 (16%)	<.001
Renal Disease	56 (21%)	40 (29%)	16 (12%)	<.001
Coronary Artery Disease	52 (19%)	34 (25%)	18 (13%)	.020
Previous Ischemic Stroke (Before Cancer Diagnosis)	43 (16%)	39 (29%)	4 (3%)	<.001
Vascular Disease	39 (14%)	25 (18%)	14 (10%)	.080
Hypothyroidism	28 (10%)	16 (12%)	12 (9%)	.560
Congestive Heart Failure	13 (4%)	11 (8%)	2 (2%)	.020
CHA_2_DS_2_-VASc (groups)				
Male < 2	105 (39%)	40 (29%)	65 (48%)	<.001
Female < 3				
Male >= 2	167 (61%)	96 (71%)	71 (52%)	
Female >= 3				
HAS-BLED (groups)				
< 3	180 (66%)	75 (55%)	105 (77%)	< .001
>= 3	92 (34%)	61 (45%)	31 (23%)	
Anticoagulation Therapy	39 (14%)	24 (18%)	15 (11%)	.190
Antiplatelet Therapy	68 (25%)	39 (29%)	29 (21%)	.170
Death (at the time of study conclusion)	148 (54%)	103 (76%)	45 (33%)	< .001

Patients were 63± 13.06 years of age and predominantly females (57%). The four most common cancer sites encountered were gastrointestinal tract (24%), breast (15%), lung and pleura (15%), and urological (13%) tumors. In addition, 90% of patients had at least one concomitant comorbidity and 12% of the patients who had Stroke/ TIA had a second Stroke/TIA within one year of the first event.

Univariate analyses (Table 2) revealed significant associations among the following variables comparing Stroke/TIA to their controls, respectively: being on active cancer-specific treatment (44% vs. 25%, *p*-value = 0.002) which had a matched OR (95% CI) of 2.9 (1.36 – 3.89) representing higher odds of being on active treatment in Stroke/TIA patients of the discordant pairs, having AF (14% vs. 4%, *p*-value = 0.007) with a matched OR (95% CI) of 4.25 (1.39 - 17.36), having at least one comorbidity (96% vs. 85%, *p*-value = 0.002) with a matched OR (95% CI) of 5 (1.68 - 20.12), having hypertension (65% vs. 52%, *p*-value = 0.030) with a matched OR (95% CI) of 1.86 (1.09 - 5.31), having diabetes mellitus (47% vs. 35%, *p*-value = 0.040) with a matched OR (95% CI) of 1.77 (1.05 – 2.99), having dyslipidemia (34% vs. 16%, p-value < 0.001) with a matched OR (95% CI) of 3.67 (1.75 - 7.66), having renal disease (29% vs. 12%, *p*-value < 0.001) with a matched OR (95% CI) of 4 (1.84 - 8.68), having a history of previous ischemic stroke before cancer diagnosis (29% vs. 3%, *p*-value < 0.001) with a matched OR (95% CI) of 9.75 (3.48 - 27.25), having coronary artery disease (25% vs. 13%, *p*-value = 0.020) with a matched OR (95% CI) of 2.23 (1.16 - 4.29), and having congestive heart failure (8% vs. 2%, *p*-value = 0.020) with a matched OR (95% CI) of 5.5 (1.2 - 51.07). In addition, univariate analyses revealed significant associations among CHA_2_DS_2_-VASc and HAS-BLED scores. Proportions of patients who had a CHA_2_DS_2_-VASc score above or equal 2 in males and above or equal 3 in females were 71% of Stroke/TIA patients vs. 52% of their controls, *p*-value < 0.001 with a matched OR (95% CI) of 3.08 (1.61 – 5.91). Proportions of patients who had HAS-BLED score above or equal 3 were 45% of Stroke/TIA patients vs. 23% of their controls, *p*-value < 0.001 with a matched OR (95% CI) of 4.33 (2.1 - 8.95). Finally, proportions of patients who died at the time of study conclusion were 76% in Stroke/TIA patients vs. 33% in their controls, *p*-value < 0.001 with a matched OR (95% CI) of 9.4 (3.74 - 23.64).

The role of CHA_2_DS_2_-VASc score was further analyzed only in patients who did not have AF. In the patients who had stroke/TIA without AF, 77 (68.1%) patient had a score above or equal 2 in males and above or equal 3 in females while in the patient who did not have stroke/TIA and were not to know to have AF, 56 (49.1%) patients had a similar score, *P*-value = 0.001.

**Fig. 1:**
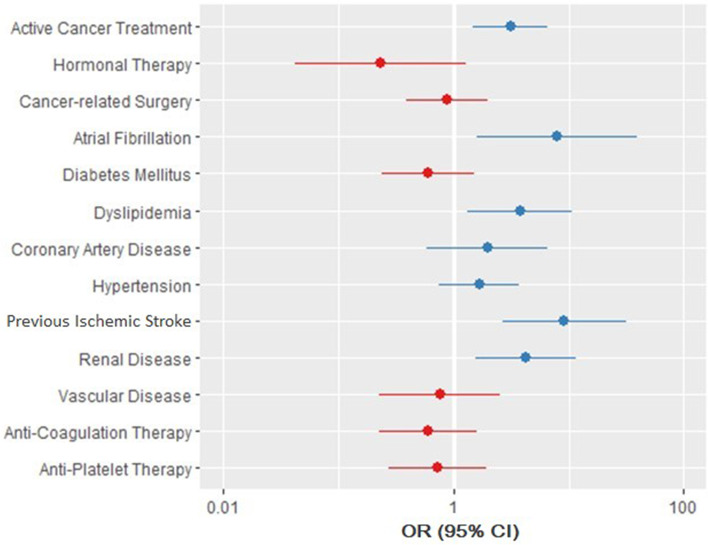
Forest Plot of Analysis of Multiple Clinical Variables and the Likelihood of stroke/TIA Occurring in Cancer Patients. Multivariate analysis was carried out using conditional logistic regression to study the effects of the clinical variables on the likelihood of having Stroke/TIA. The points represent the Odds Ratio, and the lines represent the 95% confidence interval. Points in blue represent an OR >1 indicating that cases with Stroke/TIA are more likely to have the corresponding clinical characteristic. Lines (CIs) which do not cross the null value of 1 represent statistical significance

Multivariate analysis was carried out using conditional logistic regression to study the effects of the aforementioned variables on the likelihood of having Stroke/TIA. Congestive heart failure was excluded from the model due to low sample size. The regression model was statistically significant with a likelihood test of χ2 (13) = 70.04, *p*-value < 0.001 and a concordance of 0.78 (SE = 0.05). The following variables were statistically significant: active cancer treatment (*p*-value = 0.003), AF (*p*-value = 0.010), dyslipidemia (*p*-value = 0.010), renal disease (*p*-value = 0.005), and history of previous ischemic stroke before cancer diagnosis (*p*-value < 0.001). All these variables increased the risk of developing Stroke/TIA with history of previous ischemic stroke (OR = 9.18, 95% CI = 2.66 - 31.74) and AF (OR = 7.93, 95% CI = 1.6 - 39.18) having the higher odds (Table 3) (Fig. 1).

**Table 3:** Conditional logistic regression model of stroke/TIA risk factors

Variable	B	S.E.	Wald	Odds Ratio	95% CI for Odds Ratio	***p***-value
Active Cancer Treatment at The Time of Event (Stroke/TIA)	1.13	0.39	8.7	3.11	1.46 - 6.62	.003
History of Hormonal Therapy	-1.46	0.88	2.8	0.23	0.04 - 1.29	.100
History of Cancer-Related Surgery	-0.13	0.42	0.09	0.88	0.39 - 1.99	.760
Atrial Fibrillation (New onset AF or baseline AF)	2.07	0.82	6.4	7.93	1.6 - 39.18	.010
Coronary Artery Disease	0.68	0.62	1.2	1.97	0.59 - 6.6	.270
Diabetes	-0.50	0.47	1.1	0.6	0.24 - 1.52	.280
Dyslipidemia	1.33	0.54	6.1	3.78	1.32 - 10.82	.010
Hypertension	0.51	0.41	1.5	1.67	0.74 - 3.74	.220
Vascular Disease	-0.28	0.61	0.21	0.76	0.23 - 2.51	.650
Renal Disease	1.48	0.51	8.0	4.25	1.55 - 11.63	.005
Previous Ischemic Stroke (Before Cancer Diagnosis)	2.22	0.63	12.3	9.18	2.66 - 31.74	<.001
Anticoagulation Therapy	-0.52	0.5	1.1	0.6	0.23 - 1.58	.300
Antiplatelet Therapy	-0.32	0.49	0.41	0.73	0.28 - 1.92	.520

**Fig. 2:**
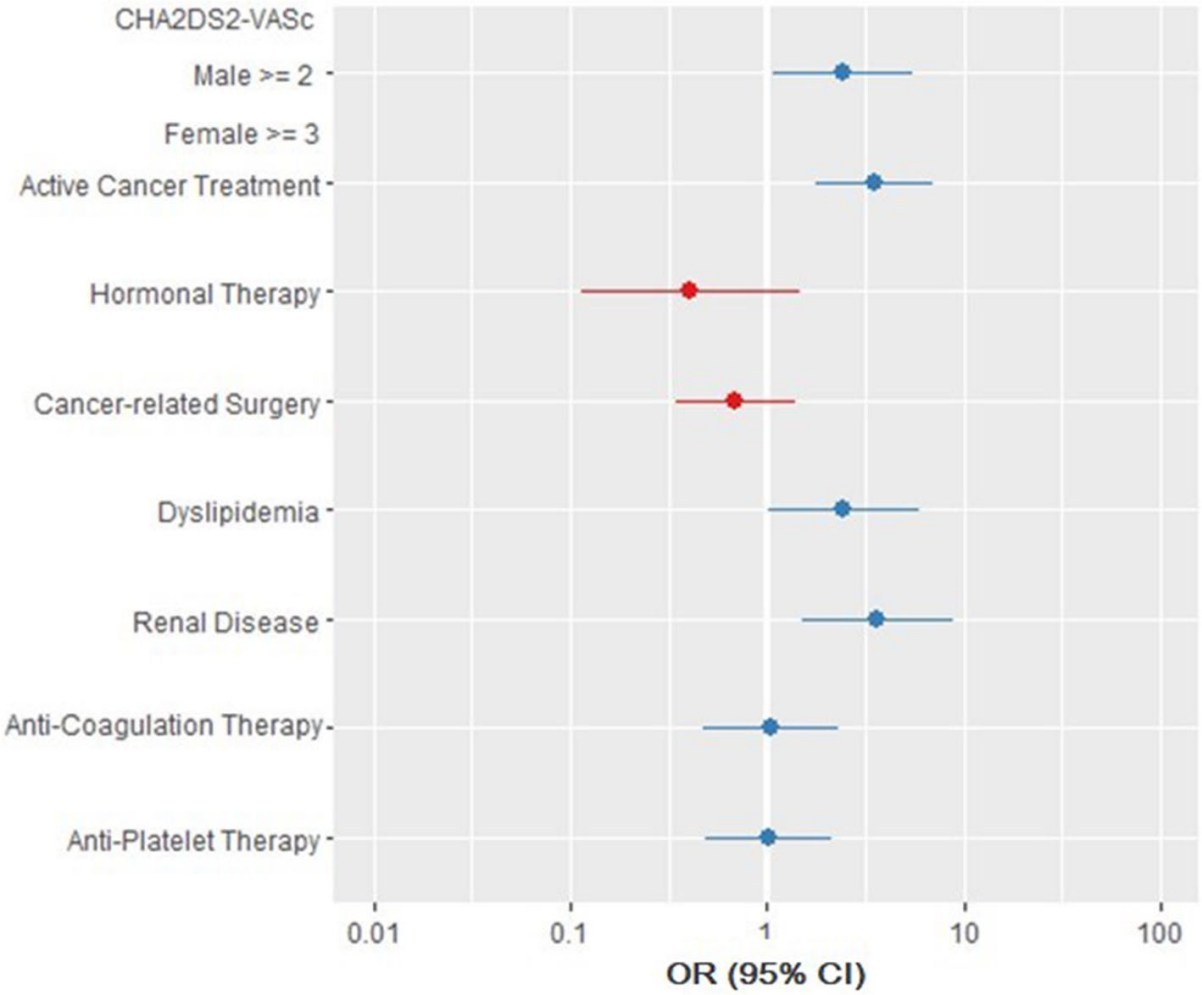
Forest plot representing CHA_2_DS_2_-VASc score contribution to Stroke/TIA in cancer patients. Another conditional logistic regression model was built to assess the effect of the CHA_2_DS_2_-VASc score on the likelihood of having Stroke/TIA in cancer patients. Variables included in computing the CHA_2_DS_2_-VASc score were excluded from the model. A CHA_2_DS_2_-VASc score of >=2 in males and >=3 in females significantly increases the risk of Stroke/TIA in cancer patients independent of AF diagnosis. The points represent the Odds Ratio, and the lines represent the 95% confidence interval. Points in blue represent an OR >1 indicating that cases with Stroke/TIA are more likely to have the corresponding clinical characteristic. Lines (CIs) which do not cross the null value of 1 represent statistical significance

In addition, another conditional logistic regression model was built to assess the effect of the CHA_2_DS_2_-VASc score on the likelihood of having Stroke/TIA in cancer patients. (Figure 2) Variables included in computing the CHA_2_DS_2_-VASc score were excluded from the model. The model was statistically significant with a likelihood test of χ2 (8) = 46.85, *p*-value < 0.001 and a concordance of 0.71 (SE = 0.06). The following variables significantly increased the risk of Stroke/TIA: having a CHA_2_DS_2_-VASc score of >=2 in males and >=3 in females (*p*-value = 0.030), active cancer treatment (*p*-value < 0.001), dyslipidemia (*p*-value = 0.046), and renal disease (*p*-value = 0.003) (Fig. 2).

## Discussion

Although the association between AF and cancer has been extensively studied before [5, 9–13], the association between AF and Stroke/TIA in the setting of cancer, on the contrary, has not. Several studies have investigated the risk factors for developing Stroke/TIA in cancer and non-cancer patients and found that classical risk factors and stroke patterns were comparable in both groups of patients. However, up to our knowledge, this is the first study that compared cancer patients who developed Stroke/TIA to a control group of cancer patients without Stroke/TIA with the same age, gender, type of cancer and were managed at the same facility within the same time frame [16–20].

We hypothesized that since both AF and cancer are independently associated with an increased risk of stroke, this risk should increase in the setting of concurrent AF and cancer. In our study, we retrospectively assessed all consecutive patients who developed ischemic Stroke/TIA at our institution over the span of 5 years; and found that among the 136 patients who developed Stroke/TIA, 14 % had AF (new onset or at baseline) which significantly and independently correlates to the likelihood to develop Stroke/TIA in cancer patients. Similar findings in a large cohort study by Hu et al showed that cancer patients who developed AF, compared to those who did not, had a significantly higher risk of thromboembolism including strokes [9].

The correlation between the CHA_2_DS_2_-VASc score and stroke risk was previously described by Patell and his colleagues [10]. They concluded that in cancer patients with preexisting AF, a higher CHADS2 and CHA_2_DS_2_-VASc score were both associated with increased risk of ischemic stroke. In our study, we included consecutive cancer patients who developed Stroke/TIA regardless of their AF status, cancer type, duration, or stage; and we assessed the utility of CHA_2_DS_2_-VASc to predict Stroke/TIA in all patients even if they didn’t have any evidence of documented AF. Moreover, we included the CHA_2_DS_2_-VASc score instead of the CHADS_2_ score given its increasing importance in the assessment of stroke risk in patients with AF. We hypothesized that since many components of the CHA_2_DS_2_-VASc score such as heart failure, hypertension, old age, and diabetes mellitus were found to be associated with stroke in the general population [21, 22]; this score would predict the risk of stroke in cancer patients regardless of the presence of AF. We found that proportions of patients who had Stroke/TIA and a high CHA_2_DS_2_-VASc score (of >=2 in males and >=3 in females) regardless of AF diagnosis are larger than those of their pairs in the control group who did not develop Stroke/TIA (71% vs. 52%, *p*-value < 0.001 with a matched OR (95% CI) of 3.08 (1.61 – 5.91). In addition to the CHA_2_DS_2_-VASc score, renal disease, dyslipidemia, and being on active cancer treatment significantly predict Stroke/TIA in cancer patients. These findings will raise questions regarding the benefit of adding more variables to the CHA_2_DS_2_-VASc score or the possibility of inventing another scheme that can predict Stroke/TIA in cancer patients to apply preventive measures.

In our study, we found that those with a history of previous ischemic stroke or AF have higher odds of developing Stroke/TIA independently from many other factors that are attributed to cancer itself. This finding is unique among many studies where atherosclerosis and vascular risk factors remain the most common risk factors of stroke in cancer and non-cancer patients [16, 17].

Finally, we found that cancer patients who developed Stroke/TIA when compared to patients without Stroke/TIA are more likely to die after an Stroke/TIA diagnosis. Similar findings were concluded before by several clinical studies [2, 8, 23, 24] stating that the prognosis may be worse in patients with cancer and stroke, due to reduced general health and the prognosis of cancer itself. This reflects the need for clinicians to be aware of the impact of these events on mortality.

Based on these results, prevention of Stroke/TIA in cancer patients should be based on assessing these risk factors. Preventive measures should be considered while accounting for the increased risk of bleeding, as our study found that patients in the Stroke/TIA group had a significantly higher HAS-BLED score. To date, a specific approach to prevent Stroke/TIA in cancer patients has not yet been established; demanding the need for future studies to prospectively validate these findings and establish a cancer-specific stroke prediction score.

### Study limitations

Our study is subject to the same limitations as other retrospective studies involving chart-reviewing due to the nature of the data entered into the electronic medical records which was not pre-designed for clinical research purposes. However, special algorithms and databases were created, and special care was taken in collecting and reviewing the data by our team. Another limitation in our study is the relatively small number of patients, despite the inclusion of all patients who developed Stroke/TIA over a period of 5 years. This is attributed to restricting the inclusion of patients only to those subjected to sufficient evaluation which concluded a definitive diagnosis of ischemic Stroke/TIA. Furthermore, patients with a vague diagnosis or patients with brain hemorrhage or brain metastasis were excluded.

## Conclusion

This is a single-center matched case-control study of cancer patients who developed Stroke/TIA with their matched cancer patients without Stroke/TIA. We found that having new-onset or history of AF, previous ischemic stroke, active cancer treatment, dyslipidemia, and kidney disease are independent risk factors for Stroke/TIA in cancer patients. In addition, having a high CHA_2_DS_2_-VASc score of >=2 in males and >=3 in females significantly increases the risk of Stroke/TIA in cancer patients independent of AF diagnosis. These patients are more likely to have to have AF at the time of event and to die earlier than their matched controls.

## Supplementary Information

**Additional file 1.**

## Data Availability

The datasets used and/or analyzed during the current study are available from the corresponding author on reasonable request. This is subject to the institutional data sharing policy.

## Abbreviations

CVD: Cerebrovascular Disease
AF: Atrial Fibrillation
Stroke: Acute Ischemic Stroke
TIA: Transient Ischemic Attack
