# Supplementary material for: Atrial fibrillation in cancer patients who develop stroke
Source: Cardiooncology. 2022 May 18;8:12. doi: 10.1186/s40959-022-00137-y (PMC9116009; doi:10.1186/s40959-022-00137-y)
Supplement: Supplementary file 1 — Additional file 1. [file 40959_2022_137_MOESM1_ESM.docx]

**Supplemental Appendix**

**Definitions of variables:**

• AF was defined as either new-onset AF or having a history of (baseline) AF.

• Active treatment was defined as a documentation of active chemotherapy, radiotherapy, or hormonal therapy.

• Cancer-related surgery was defined as procedures requiring general anesthesia targeted towards cancer management.

• Cancer stage was defined according to as the following criteria:

- Localized: Cancer is limited to the site of origin without invasion or distant metastasis.

- Regional: Cancer with local invasion or with spread to regional lymph nodes.

- Distant: Cancer with spread to distant lymph nodes or with metastasis to another organ.

- Hematological cancers, intracranial cancers, or cancers with unknown stage or were defined as “unstageable”.

• Renal disease was defined as a documented diagnosis of CKD or AKI, or as elevated Cr above baseline.

• Liver disease was defined as chronic hepatitis, acute hepatitis, or cirrhosis.

• Vascular disease was defined based on CHA2DS2-VASc criteria: myocardial infarction, percutaneous coronary intervention, coronary artery bypass surgery or peripheral arterial disease.

• Anti-platelet usage was defined as being on ASA or clopidogrel.

• Anti-coagulation usage was defined as taking one of the following agents: warfarin, DOACs, LMWH, or heparin.
